# KNOWLEDGE AND ATTITUDES TOWARD LDCT LUNG CANCER SCREENING AND SMOKING AMONG AFRICAN AMERICANS: A MIXED METHODS STUDY

**DOI:** 10.1093/geroni/igz038.681

**Published:** 2019-11-08

**Authors:** Tung-Sung Tseng

**Affiliations:** Tulane University, New Orleans, Louisiana, United States

## Abstract

The purpose of this study is to investigate knowledge, attitudes, and smoking cessation needs for African Americans who receive Low Dose Computed Tomography (LDCT) in an effort to reduce the health burden of lung cancer. A mixed method study was conducted among African Americans who received LDCT. The sample size for both the quantitative and qualitative approach was fifteen. The results showed that 73% of participants were male, the mean age was 61.8(SD=4.6) years old. Smoking history was long but 64% of the patients had a low nicotine dependence. Participants had a moderate/lower knowledge score (Mean=4.3 SD=2.6), and most had a positive attitude. Similar findings were also observed in the qualitative analysis. Understanding the factors associated with smoking cessation among at-risk African American smokers will help reduce disparities in lung cancer burden, and is important to improve health for medically underserved minority populations.

